# LIGHT THERAPY FOR SLEEP DISTURBANCE OF NURSING FACILITY RESIDENTS WITH DEMENTIA: A SYSTEMATIC REVIEW

**DOI:** 10.1093/geroni/igac059.2801

**Published:** 2022-12-20

**Authors:** Wenxin Bian, Mark Toles, Rose Mary Xavier

**Affiliations:** University of North Carolina at Chapel Hill, Chapel Hill, North Carolina, United States; UNC Chapel Hill, Chapel Hill, North Carolina, United States; University of North Carolina at Chapel Hill, Chapel Hill, North Carolina, United States

## Abstract

**Background:**

Bright light therapy has shown promise in addressing sleep problems in nursing facility residents with dementia. However, recent studies yielded conflicting outcomes and few studies focused on nursing facilities. The purpose of this systematic review was to describe effectiveness of light interventions in nursing facility residents with dementia. Method: We searched PubMed, CINAHL, EMBASE, PsychINFO, and Scopus using key terms “sleep”, “dementia” and “residential facilities”, and synthesized data with thematic analysis and vote-counting.

**Results:**

Of eight studies that met inclusion criteria, six were randomized controlled trials and 2 were quasi-experimental. Sample size ranged from 11 to 77 residents. Studies tested 3 light therapies: timed bright light (n=6), timed regular light (n=1), and variable 24hour light (n=1). Light delivery method, light exposure, and adherence to therapy protocols were not consistently reported. All studies indicated light therapy improved some resident outcomes, such as sleep efficacy and total sleep time; however, 88% of studies did not report sampling strategies or a statistical power analysis and 22% had small sample size (n < 15).

**Conclusion:**

Insufficient evidence is available to recommend light therapies for nursing facility residents with dementia. Adequately statistically powered studies that are rigorously designed with representative samples are needed for robust estimation of the effects of light therapy on sleep. Future studies must account for the unique characteristics of nursing facility residents with dementia that impact their adherence to light therapy.

